# Evaluation of Oral Tebipenem as a Step-Down Therapy following Intravenous Ertapenem against Extended-Spectrum β-Lactamase-Producing Escherichia coli in a Hollow-Fiber *In Vitro* Infection Model

**DOI:** 10.1128/aac.00908-22

**Published:** 2023-02-09

**Authors:** B. D. VanScoy, S. Jones, H. Conde, L. V. Friedrich, N. Cotroneo, S. M. Bhavnani, P. G. Ambrose

**Affiliations:** a Institute for Clinical Pharmacodynamics, Inc., Schenectady, New York, USA; b Spero Therapeutics, Inc., Cambridge, Massachusetts, USA

**Keywords:** tebipenem, ertapenem, step-down oral therapy, *in vitro*, hollow-fiber *in vitro* infection model, *Escherichia coli*, extended-spectrum β-lactamase

## Abstract

Tebipenem is an orally bioavailable carbapenem in development for the treatment of patients with complicated urinary tract infections. Herein, we describe the results of studies designed to evaluate tebipenem’s potential as an oral (p.o.) transition therapy from intravenous (i.v.) ertapenem therapy for the most common uropathogen, Escherichia coli. These studies utilized a 7-day hollow-fiber *in vitro* infection model and 5 extended-spectrum β-lactamase-producing E. coli challenge isolates. Human free-drug serum concentration-time profiles for tebipenem 600 mg p.o. every 8 h and ertapenem 1 g i.v. every 24 h were simulated in the hollow-fiber *in vitro* infection model. Samples were collected for bacterial density and drug concentration determination over the 7-day study period. Generally, ertapenem monotherapy resulted in a greater reduction in bacterial density than did tebipenem monotherapy. In the treatment arms in which ertapenem dosing was stopped following dosing for 1 or 3 days, immediate bacterial regrowth occurred and matched that of the growth control. Finally, in the treatment arms in which ertapenem dosing was stopped following dosing for 1 or 3 days and tebipenem dosing was initiated for the remainder of the 7-day study, the intravenous-to-oral transition regimen reduced bacterial burdens and prevented regrowth. Given that transition from intravenous to oral antibiotic therapy has been shown to reduce hospital length of stay, nosocomial infection risk, and cost, and improve patient satisfaction, these data demonstrate tebipenem’s potential role as an oral transition agent from intravenous antibiotic regimens within the antibiotic stewardship paradigm.

## INTRODUCTION

The transition from intravenous to oral antibiotic therapy has many benefits, including reducing the length of hospital stay, lowering the risk of nosocomial infections, and reducing overall costs (1–4). Moreover, early hospital discharge on oral antibiotic therapy is associated with high patient satisfaction about the quality of hospital care received (5). For these reasons, antibiotic stewardship teams have long implemented programs designed to identify candidate patients for transition from intravenous to oral antibiotic therapy (6, 7).

Fluoroquinolones and trimethoprim-sulfamethoxazole (TMP-SMX) are commonly utilized when transitioning patients from intravenous to oral antibiotic therapy. However, safety concerns (8) and the percentages of fluoroquinolone-resistant *E. coli* in the USA and Europe, which approach 30%, and the percentages of fluoroquinolone-resistant *K. pneumoniae*, which are 10 to 15% and up to 40% in the USA and Europe, respectively, (9–11) have made this antibiotic class an increasingly unsuitable therapeutic option. Additionally, TMP-SMX resistance among extended-spectrum β-lactamase (ESBL)-producing *Enterobacterales* isolates has been reported to be as high as 30%, and fluoroquinolone-TMP-SMX cross-resistance among ESBL-producing isolates is common (9, 12).

Tebipenem, an orally bioavailable carbapenem which is administered as a prodrug (tebipenem pivoxil hydrobromide), is in development for the treatment of patients with complicated urinary tract infections. Tebipenem has broad *in vitro* spectrum activity, which includes *Enterobacterales* species commonly associated with complicated urinary tract infections and acute pyelonephritis, including those that are fluoroquinolone or TMP-SMX resistant and/or produce ESBL (13, 14). The objective of these studies was to use a 7-day hollow-fiber *in vitro* infection model to evaluate the potential of oral tebipenem as a transition therapy from ertapenem intravenous therapy against ESBL-producing *E. coli.*

## RESULTS

### Susceptibility test results.

The tebipenem and ertapenem MIC values and those for other agents of interest, sequence type, and associated known resistance determinants for the isolates used in the hollow-fiber *in vitro* infection model studies are presented in Table 1. All five isolates were ESBL positive and were resistant to ceftriaxone, levofloxacin, and TMP-SMX. Furthermore, four of the five isolates studied were sequence type 131 (ST131).

**TABLE 1:** Tebipenem and ertapenem MIC values and their associated known resistance determinants for the isolates utilized in the hollow-fiber *in vitro* infection model studies

E. coli isolate	Resistance mechanism(s)	Sequence type, serogroup	Modal MIC (mg/L)				
			Tebipenem	Ertapenem	Ceftriaxone	Levofloxacin	TMP-SMX^*a*^
NCTC 13441	CTX-M-15	ST131	0.015	0.03	>16	>4	>16
4643	CTX-M-15, OXA1/30	ST131	0.008	0.015	>16	>4	>16
1033345	CMY-2, TEM-1	Unknown	0.06	0.25	>16	>4	>16
998822	CTX-M-15, OXA1, OXA-30	ST131, O25b	0.03	0.015	>16	>4	>16
13319	CTX-M-15, AcrAB-TolC, OmpC	ST131, O25b	0.015	0.125	>16	>4	>16

a TMP-SMX, trimethoprim-sulfamethoxazole.

### Pharmacokinetic studies.

**FIG 1:**
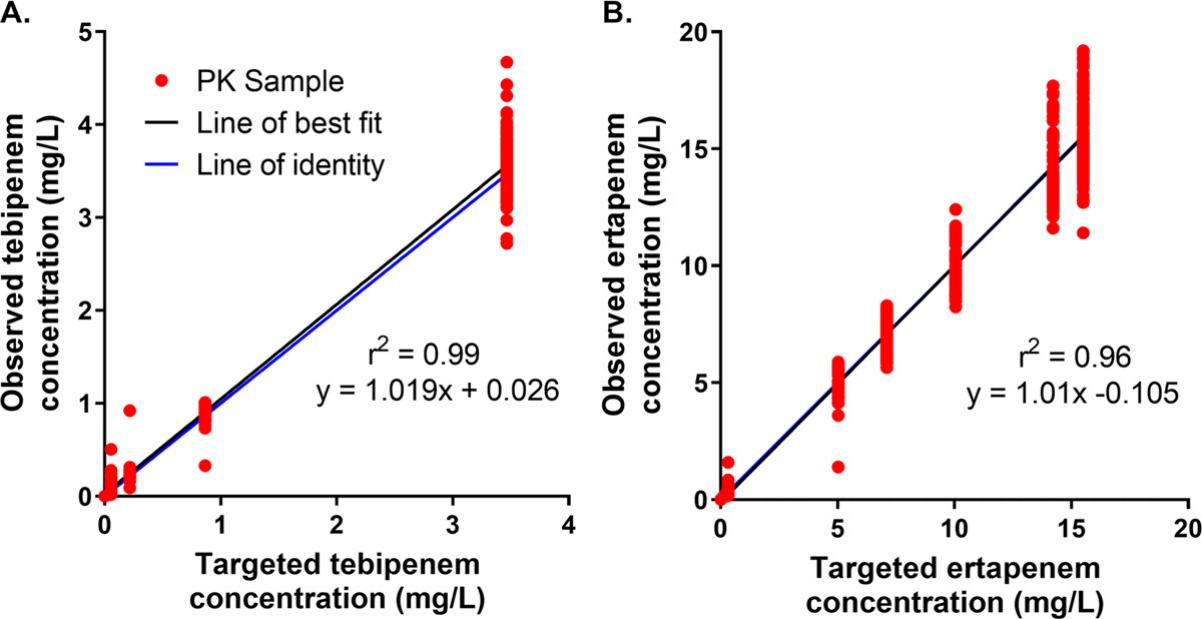
Relationship between targeted and observed tebipenem (A) and ertapenem (B) concentrations simulated in the hollow-fiber *in vitro* infection model studies.

**FIG 2:**
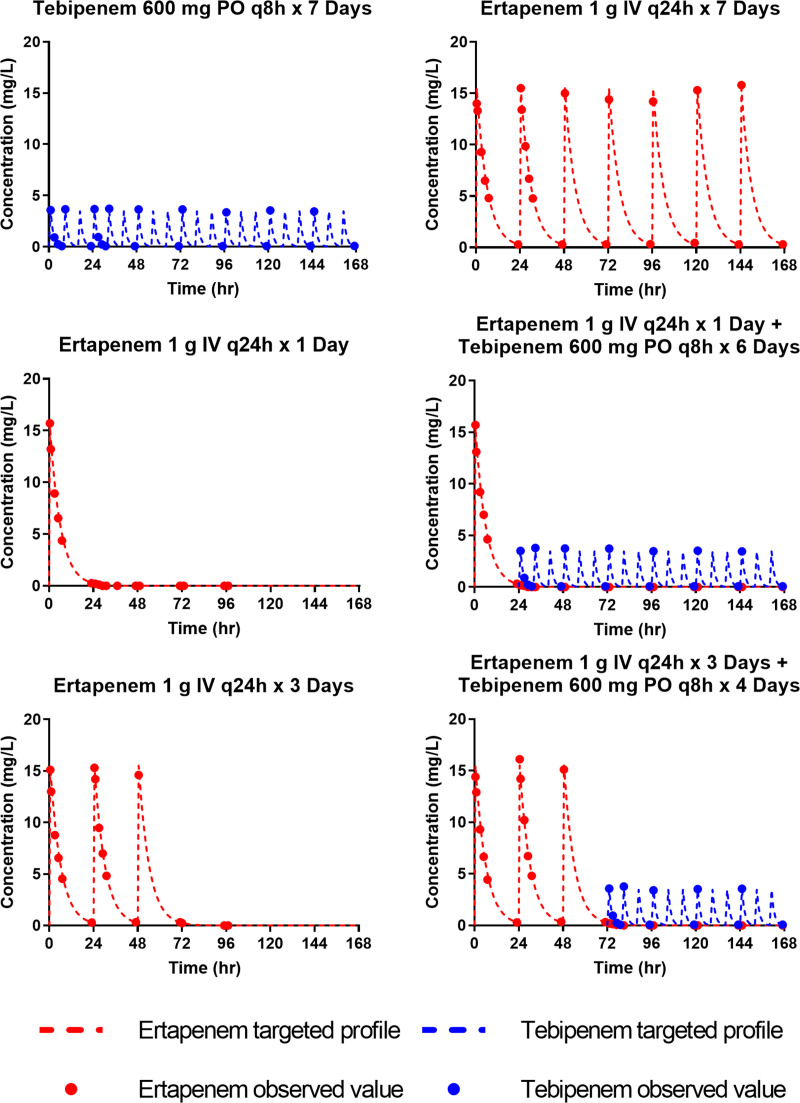
Targeted free-drug concentration time profiles for tebipenem and ertapenem in the hollow-fiber *in vitro* infection model studies, with observed data overlaid for a sample of evaluated regimens.

As shown in Fig. 1, there was good agreement between the targeted and observed tebipenem (Fig. 1A) and ertapenem (Fig. 1B) concentration-time profiles simulated in the hollow-fiber *in vitro* infection model. As demonstrated by the high coefficients of determination for these data (tebipenem, *r*^2^ = 0.99; ertapenem, *r*^2^ = 0.96), the precision was excellent. The slopes of 1.02 for tebipenem and 1.01 for ertapenem demonstrated minimal bias. The observed concentration-time profiles simulated in the hollow-fiber *in vitro* infection model for each of the dosing regimens evaluated over the 7-day period are shown in Fig. 2.

### Hollow-fiber *in vitro* infection model treatment studies.

**FIG 3:**
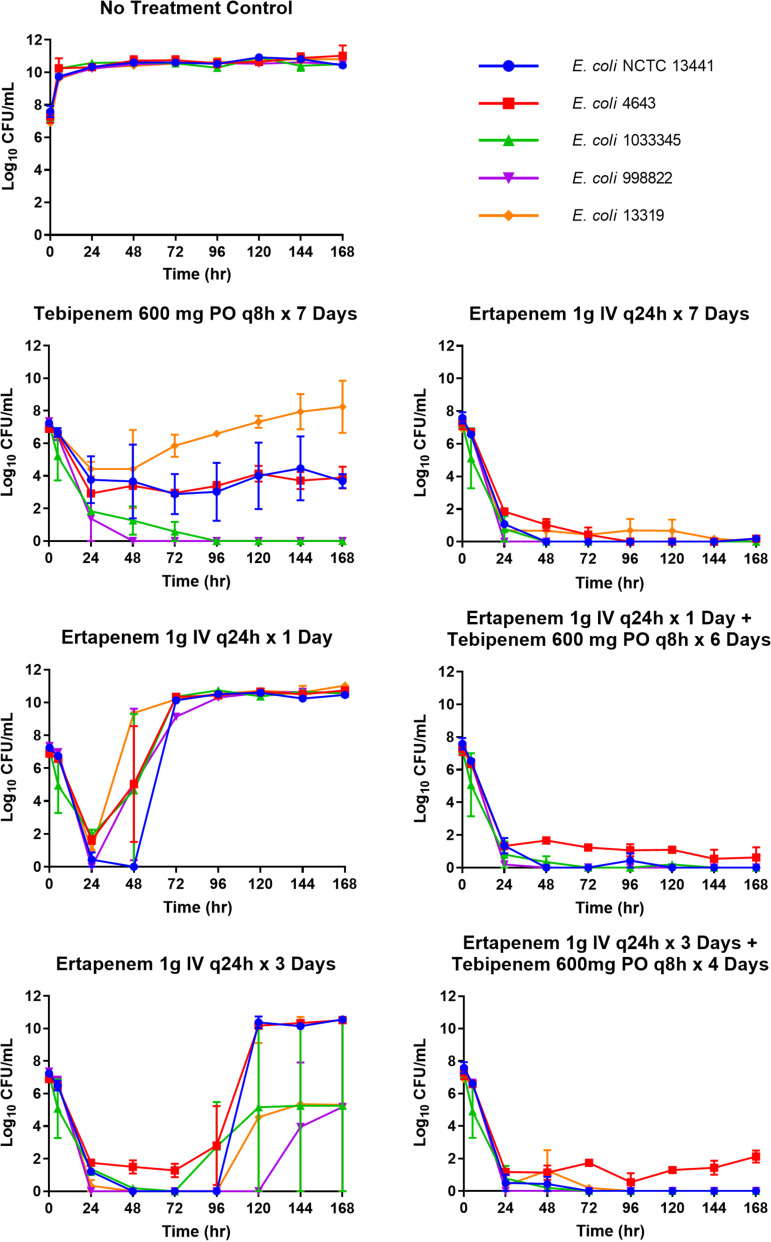
Average bacterial burdens observed for five E. coli isolates in the 7-day hollow-fiber *in vitro* infection model studies.

The average results of the 7-day hollow-fiber *in vitro* infection model studies are shown in Fig. 3. All five challenge isolates grew well in the no-treatment control group and reached densities of >10^10^ CFU/mL by 24 h. A monotherapy regimen consisting of tebipenem 600 mg p.o. every 8 h successfully reduced bacterial burdens below those initially inoculated over 7 days for four out of the five isolates evaluated. The lone exception was E. coli 13319. The bacterial densities of E. coli 1033345 and E. coli 998822 were reduced to the level of detection by 48 to 96 h without regrowth over 7 days. The bacterial densities of E. coli NCTC 13441 and E. coli 4643 were reduced by 2.5 to 3 log_10_ CFU by 24 h and generally remained so for the 7-day duration of the study.

The 7-day regimen consisting of ertapenem 1 g intravenous (i.v.) every 24 h successfully reduced the bacterial burdens below those initially inoculated in the hollow-fiber *in vitro* infection model over the 7-day study period for all five E. coli isolates. In contrast, the 1- and 3-day ertapenem regimens consisting of 1 g every 24 h failed to suppress bacterial growth at 24 to 48 h and 72 to 96 h from dosing initiation. However, when the oral tebipenem regimen with 600 mg every 8 h followed the 1- and 3-day ertapenem regimens, bacterial burdens were suppressed for the entire 7-day duration of the study. Moreover, no amplification of resistant subpopulations was noted for any of the isolates.

## DISCUSSION

The objective of these studies was to use a 7-day hollow-fiber *in vitro* infection model and a study design sufficient to evaluate tebipenem’s potential as an oral transition agent from ertapenem intravenous therapy against ESBL-producing E. coli.

The study design strengths included the presence of not only a no-treatment control arm but also ertapenem 1- and 3-day control arms. These additional control arms allowed for the confirmation that short drug exposure durations were insufficient to maintain bacterial growth suppression over the 7-day study period. In treatment arms for which ertapenem dosing was stopped following dosing for 1 or 3 days and tebipenem dosing was initiated for the remainder of the 7-day study, the intravenous-to-oral transition regimen reduced bacterial burdens and prevented regrowth of all 5 challenge isolates. Additionally, we evaluated multiple clinical isolates in an effort to better describe the variability of effect that may be encountered among isolates in the clinic. These isolates represented clinically relevant, problematic pathogens based on sequence type, ESBL production, and resistance phenotypes (quinolone resistance and TMP-SMX resistance).

These study results have important implications for clinicians and the health care system, as well as for patients. A primary tenet of antimicrobial stewardship programs is to minimize the unintended consequences of antimicrobial therapy (15, 16). One way to accomplish this is to decrease the length of stay via transitioning patients from intravenous to oral antibiotics once they are clinically stable. Antibiotic stewardship teams have long implemented programs designed to identify candidate patients for transition from intravenous to oral antibiotic therapy (6, 7). Typically, these criteria include the ability to take oral medications, the abetment of infection signs and symptoms, normalization of the white blood cell count, and patients being afebrile (17–19). In fact, this concept is considered a strong recommendation in the *IDSA Stewardship Guidelines* (16), thus providing further support for this approach. Transitioning from intravenous to oral therapy allows for early patient discharge and has been demonstrated to decrease the overall cost of care through better management of resources (e.g., facility, personnel, drug, and other supplies) and patient flow and by minimizing the patient’s risk of nosocomial infection (17, 20). Moreover, it increases patient satisfaction with the quality of hospital care received (5).

With regard to the tebipenem and ertapenem 7-day treatment arms, there were notable differences among the E. coli isolates in the response to drug exposure. The 7-day intravenous ertapenem regimen successfully reduced the bacterial burdens to near the level of detection in the hollow-fiber *in vitro* infection for all five E. coli isolates, while the 7-day oral tebipenem regimen did so for two of the five isolates studied, with at least a 2-log_10_ CFU reduction from baseline for another two isolates. Of interest is the single isolate, E. coli 13319, for which tebipenem failed to maintain a reduction in the bacterial burden below that initially inoculated over 7 days. Possible explanations for this observation include efflux pump expression upregulation, porin expression downregulation, and/or penicillin-binding protein mutations in response to tebipenem exposure. As shown in Table 1, among the known resistance mechanisms associated with E. coli 13319 is the presence of the OmpC gene and the AcrAB-TolC efflux pump protein. Either or both resistance determinants may explain the observations noted with E. coli 13319.

There are limitations to the study described here. Perhaps most importantly, we utilized a 7-day hollow-fiber *in vitro* infection model, which can trap β-lactamase enzymes within the peripheral model compartment. Given that the peripheral model compartment is where the microbe-drug interaction occurs, trapping β-lactamase enzymes in this compartment may result in an overestimation of the drug needed for efficacy. In addition, tebipenem was studied as an oral transition agent only for ertapenem and against E. coli. It is unknown if similar results would be obtained in combination with other intravenous antibiotic-microbial species pairs.

The transition from intravenous to oral antibiotic therapy has been shown to reduce hospital length of stay, nosocomial infection risk, and cost and improve patient satisfaction. These data demonstrate tebipenem’s potential role as a transition therapy within the antibiotic stewardship paradigm. These data also suggest the need to evaluate tebipenem as the oral transition agent from other intravenous antibiotic regimens and with other bacterial species of clinical relevance.

## MATERIALS AND METHODS

### Study antibiotic drug supply, challenge isolate panel, and *in vitro* susceptibility studies.

Tebipenem was provided by Spero Therapeutics (Cambridge, MA, USA). Ertapenem was purchased from Henry Schein (Melville, NY, USA). The challenge isolates utilized in these studies were either purchased from JMI Laboratories (North Liberty, IA) or provided by the National Collection of Type Cultures (NCTC). The challenge panel included five E. coli isolates that were selected based upon their multilocus sequence type (e.g., ST131), resistance mechanisms (e.g., ESBL production), and MIC values for tebipenem and ertapenem and confirmed resistance to other antibiotics (e.g., fluoroquinolone and TMP-SMX). Susceptibility studies were performed in accordance with the guidelines of the Clinical and Laboratory Standards Institute (21) and completed in triplicate over a 2-day period. The final MIC values were reported as modal values. Cation-adjusted Mueller-Hinton broth (BD Laboratories, Franklin Lakes, NJ, USA) was utilized in all susceptibility studies.

### Hollow-fiber *in vitro* infection model and sample collection and processing.

The hollow-fiber *in vitro* infection model utilized in the studies described herein has been previously described (22, 23). The hollow-fiber cartridge is comprised of a peripheral compartment and a central compartment, which are separated by a semipermeable membrane. Bacteria were inoculated into the peripheral compartment, from which they could not escape into the central compartment due to the semipermeable membrane aperture size. However, growth medium drugs and bacterial waste products freely passed into and out of the peripheral compartment via the apertures. Peristaltic pumps were used to infuse new growth medium through the hollow-fiber cartridge. Study drug was introduced into the central compartment using a computer-controlled pump and continuously diluted using drug-free medium and a peristaltic pump to mimic the desired drug elimination rate. Fresh medium was infused into the central compartment such that the desired concentration-time profile could be generated within the peripheral compartment. Samples were collected for bacterial density and drug concentration determination over the study period. In the studies described here, bacterial inoculants were prepared from cultures grown overnight to mid-log phase on plates containing Trypticase soy agar plus 5% sheep blood (BD Laboratories) at 35°C. Subsequently, the colonies were suspended in flasks containing cation-adjusted Mueller-Hinton broth medium and set in a 35°C shaking water bath; the optical density at 630 nm (OD_630_) was determined utilizing a confirmed growth curve specific to each challenge isolate. The inoculates were centrifuged at 5,000 rpm; subsequently, the supernatant was removed, resuspended with fresh Mueller-Hinton broth, and then diluted to a final concentration of 1 × 10^7^ CFU/mL. A 10-mL bacterial suspension of each isolate was then introduced into the hollow-fiber *in vitro* infection model (FiberCell Systems, Frederick, MD) peripheral compartments.

Over the duration of the study, 1-mL samples were collected from the peripheral compartment of the hollow-fiber *in vitro* infection model (daily for 7 days) for bacterial density determination. These samples were washed twice with sterile normal saline by centrifuging at 5,000 rpm, removing the supernatant and resuspending it back to the initial total volume. Each sample was then serially diluted and quantitatively cultured on drug-free Trypticase soy agar plus 5% sheep blood agar plates. A portion of each 1-mL sample was plated onto Mueller-Hinton agar drug-containing plates (4× tebipenem MIC value; 2× ertapenem MIC value) for enumeration of the drug-resistant subpopulations. Preliminary work with ertapenem failed to allow growth on drug-supplemented agar plates, and as such, the concentration was decreased to 2× MIC.

Over the course of the study, a minimum of two 1-mL samples per day were collected from the central compartment of the hollow-fiber *in vitro* infection model for drug concentration determination. Specimens for drug concentration determination were frozen immediately and stored at −80°C until assayed.

### Study regimens.

Ertapenem free-drug concentration-time profiles simulated within the hollow-fiber *in vitro* model were based upon information available on the product label (e.g., serum half-life [4 h] and protein binding [90%]) (24). Similarly, tebipenem free-drug concentration-time profiles simulated within the hollow-fiber *in vitro* model were based upon information from a phase 1 study and were provided by Spero Therapeutics (e.g., serum half-life [1 h {25}] and protein binding [45%; Spero Therapeutics, data on file]).

The intravenous-to-oral transitional study regimens had a total duration of 7 days. In these studies, ertapenem 1 g i.v. every 24 h was administered for 1 or 3 days and followed by tebipenem 600 mg p.o. every 8 h for 6 or 4 days, respectively. The study also included no-treatment and active control regimens. The active control regimens included ertapenem 1 g i.v. every 24 h administered for 7 days and tebipenem 600 mg p.o. every 8 h for 7 days. All studies were conducted in duplicate.

### Drug assay methods.

All tebipenem and ertapenem concentrations were assayed using a chromatography-tandem mass spectrometry (LC-MS/MS) method on a Sciex 5500 system with an ExionLC AC front-end device. Drug concentrations were quantitated using an internal standard (d5-tebipenem).

The standard curve for tebipenem was generated by plotting the ratio of the peak area to the d5-SPR859 peak area versus the standard concentration. The standard curve was linear over a concentration range of 0.010 to 10.000 μg/mL, with *r*^2^ values ranging from 0.9996 to 0.9999. The lower limit of tebipenem quantification was 0.010 μg/mL. The interassay coefficient of variation (CV) percentages for the quality-control samples at tebipenem concentrations of 0.010, 0.040, 1.00, and 8.00 μg/mL were 3.96%, 2.88%, 1.45%, and 1.46%, respectively. The interassay recovery percentages for the quality-control samples at concentrations of 0.010, 0.040, 1.00, and 8.00 μg/mL were 102%, 101%, 100%, and 99.0%, respectively.

The standard curve for ertapenem was generated by plotting the ratio of the ertapenem peak area to the d5-SPR859 peak area versus the standard concentration. The standard curve was linear over a concentration range of 0.100 to 30.000 μg/mL, with *r*^2^ values ranging from 0.9983 to 0.9996. The lower limit of ertapenem quantification was 0.100 μg/mL. The interassay CV percentages for the quality-control samples at concentrations of 0.100, 0.250, 2.50, and 15.00 μg/mL were 6.35%, 3.73%, 3.89%, and 3.24%, respectively. The interassay recovery percentages for the quality-control samples at concentrations of 0.100, 0.250, 2.50, and 15.00 μg/mL were 103%, 99.0%, 100%, and 102%, respectively.
